# Performance characterization of a traditional wood‐fired pizza oven

**DOI:** 10.1111/1750-3841.16268

**Published:** 2022-08-07

**Authors:** Aniello Falciano, Paolo Masi, Mauro Moresi

**Affiliations:** ^1^ Department of Agricultural Sciences University of Naples Federico II Portici Italy; ^2^ Department for Innovation in the Biological, Agrofood and Forestry Systems University of Tuscia Viterbo Italy

**Keywords:** baking test, energy consumption, thermal efficiency, transitory and pseudosteady‐state regime performance, water heating test, wood‐fired pizza oven

## Abstract

**Abstract:**

Neapolitan pizza, a renowned Italian food recognized as one of the traditional specialties guaranteed (TSG) by European Commission Regulation no. 97/2010, should be exclusively baked in wood‐fired ovens for approximately 90 s. Despite its extensive use in restaurants and rotisserie shops all around the world, such equipment has been very poorly studied thus far. The aims of this study were to characterize the operation of a pilot‐scale wood‐fired pizza oven from its start‐up phase to its baking operation and assess its thermal efficiency. To manage brick firing, the oven was lighted at a firewood feed rate (*Q*_fw_) of 3 kg/h for just 1 h on the first day, 2 h on the second day, 4 h on the third day, and approximately 8 h on the fourth day. Independent of its lighting frequency, after 4‐6 h, the oven vault or floor temperature approached an equilibrium value of 546 ± 53°C or 453 ± 32°C, respectively. The initial oven floor temperature gradient was linearly related to *Q*_fw_, while the maximum floor temperature tended to an asymptotic value of 629 ± 43°C at *Q*_fw_ = 9 kg/h. The well‐known water boiling test was adapted to assess the heat absorbed by a prefixed amount of water when the pizza oven was operating in pseudosteady‐state conditions at *Q*_fw_ = 3 kg/h. The thermal efficiency of this oven was 13 ± 4%, and this value was further confirmed by other baking tests with four different white and tomato pizza products.

**Practical Application:**

Although wood‐fired pizza ovens are largely used all over the world, little is known about their transitory and pseudosteady‐state regime performance. This study shows how to perform the start‐up procedure of pilot‐scale equipment and, independent of the operator's ability, how to achieve pseudosteady‐state conditions using different firewood feed rates. Finally, its thermal efficiency was assessed by water heating and pizza baking tests, which allowed a rough estimation of firewood consumption.

## 1 INTRODUCTION

Neapolitan pizza is an Italian food well known in the global market. It was recognized as one of the traditional specialties guaranteed (TSG) by the European Commission Regulation No. 97/2010 (EC, 2010). Additionally, the art of the Neapolitan pizza maker (*Pizzaiuolo*) was inscribed on the Representative List of the Intangible Cultural Heritage of Humanity by the United Nations Education, Scientific and Cultural Organization (UNESCO, 2017). All its production steps (namely, preparation of dough, its rising process, ball shaping, garnishing, and baking) were fully described by Masi et al. (2015). It is worth noting that Neapolitan pizza TSG should be exclusively baked in wood‐fired ovens for approximately 90 s (EC, 2010).

Wood‐fired ovens are widely used in restaurants, rotisserie shops, and bakeries worldwide. Today, in the United States, there are approximately 77,000 pizzerias employing more than 1 million people (Kuscer, 2022), while in Italy, approximately 127,000 companies with pizzeria activities are currently operating with the help of approximately 100,000 employees (Anon, 2020). In Italy, the overall turnover of pizza is near € 15 billion per year (Anon, 2020). The production activities of artisanal pizza in restaurants, pizzerias, bars, delicatessens, and takeaway restaurants cover approximately 80% of pizza sales, with the remaining 20% being related to frozen pizza (Anon, 2020).

As a result of the widespread use of wood‐fired ovens, there is growing attention to their stack emissions since these are responsible for indoor and outdoor air pollution. The burning of wood logs or briquettes in pizzerias was in fact found to be a major source of black carbon and particulate matter with sizes smaller than 2.5 µm (PM_2.5_) within the metropolitan area of São Paulo (Brazil), one of the largest megacities in the world with more than 20 million inhabitants, 8 million vehicles, and 8000 pizzerias, approximately 6400 of which are equipped with pizza ovens fueled with approximately 48 metric tons/year of firewood (Kumar et al., 2016). The average concentration of PM_2.5_ at the exit of the oven chimney was found to be as high as 6171 µg/m^3^, while that in indoor areas was near 68 µg/m^3^ (Lima et al., 2020), a level definitively greater than the indoor 24‐h mean level (15 µg/m^3^) recommended by WHO (2018).

In the technical literature, wood‐fired ovens have been very poorly studied thus far. Igo et al. (2020) found that the thermal efficiency of a metal fired‐wood oven to heat 20 L of water from 35 to 90°C was approximately 19%, while the energy lost by hot fumes or dispersed through the oven walls was approximately 55% or 26%, respectively. The efficiency of two indirect and semidirect wood‐fired bakery ovens was assessed by measuring an overall consumption of 0.55 and 0.90 kg of wood per kg of wheat flour baked, respectively (Manhiça, 2014; Manhiça et al., 2012). Practically, no information about the thermal performance of wood‐fired pizza ovens is currently available, and this is a strong limitation in modeling mass and heat transfer mechanisms during pizza baking. In contrast, the performance of alternative electric pizza ovens in steady and unsteady operating conditions was analyzed by resorting first to a three‐dimensional numerical model (Ciarmiello & Morrone, 2016a) and second to a three‐dimensional computational fluid dynamics model to simulate radiative and convective heat transfer mechanisms (Ciarmiello & Morrone, 2016b). During pizza cooking, the decrease in the oven floor temperatures was primarily affected by wall emissivity, while the increase in pizza temperature was sensitive to pizza and wall emissivity in the ranges of 0.6–1.0 and 0.7–1.0, respectively (Ciarmiello & Morrone, 2016b).

Wood‐fired ovens generally consist of a base of tuff and fire brick covered by a circular cooking floor over which a dome made of refractory materials is built to minimize heat dispersion. Their geometric dimensions (i.e., cooking floor diameter of 105‐ −140 cm; vault height of 40–45 cm; oven mouth of 45–50 cm in width and 22–25 cm in height) allow the temperature of the cooking floor and dome to be kept at approximately 430°C and 485°C, respectively, ensuring the baking quality of the Neapolitan pizza TSG (EC, 2010).

The operation of a wood‐fired oven accounts for four interactive processes: combustion, heat, flow, and mass transfer. As firewood burns in a specific area of the baking floor, releasing energy and forming the flame, air naturally enters through the open entry door of the oven and makes firewood burn, while the resulting flue gases are discharged through the oven chimney. Heat transfer is just one such process, and no exact solution can be obtained unless four groups of equations, corresponding to all these processes, are solved simultaneously. In particular, the basic unsteady‐state energy equation of heat transfer from the flame to the oven walls and floor must include a mathematical model of heat transfer in the oven, and its solution is generally of the numerical type. Even for an approximate solution, the amount of calculation is very large, and semiempirical methods are those most often used for engineering design (Zhang et al., 2016).

The main aim of this study was to characterize the operation of a pilot‐scale wood‐fired pizza oven from its start‐up phase (according to the procedure suggested by the manufacturer) to its baking operation to provide a basis for future modeling of novel pizza oven design. The well‐known water boiling test, generally used to measure the thermal efficiency of cookstoves (Global Alliance for Clean Cookstoves, 2014), was adapted to measure the energy efficiency of the pizza oven in pseudosteady‐state conditions when heating a prefixed amount of water or different pizza types.

## 2 MATERIALS AND METHODS

### 2.1 Raw materials

To prepare the Neapolitan pizza bases used in this study, the following ingredients were used: (i) soft wheat flour type 00 with a nominal moisture content of 12% w/w was kindly supplied by Mulino Caputo (Antimo Caputo Srl, Naples, Italy), (ii) fresh brewer's yeast (Lesaffre Italia, Trecasali, Parma, Italy), (iii) Sicilian fine table salt (Italkali, Petralia, Palermo, Italy), and (iv) deionized water at 16–18°C. Each pizza base was baked as such or garnished using sunflower oil (Mepa Srl, Terzigno, Naples, Italy) and/or tomato puree at 7.0 ± 0.2 °Brix (Mutti SpA, Parma, Italy). The wood‐fired oven was fed dry, seasoned oak logs from the Royal Park of Portici (Department of Agricultural Sciences of the University of Naples—Federico II), and their average weight, length, and diameter were equal to 600 ± 200 g, 250 ± 20 mm, and 40 ± 10 mm, respectively.

### 2.2 Pizza preparation

The pizza dough was prepared by mixing 1600 g of soft wheat flour type 00 and 50 g of table salt with 1 L of deionized water at room temperature, where 1 g of fresh brewer's yeast had been previously dispersed to allow its hydration for approximately 3 min. This operation was carried out in a spiral mixer (Grilletta IM5; Famag Srl, Milan, Italy) set at level 1 for 18 min (see Figure S1). The dough was then left resting at room temperature for 20 min. Thereafter, the dough was subdivided into dough balls weighing ∼250 g each. These were placed over 60 cm × 40 cm plastic trays (Giganplast, Monza and Brianza, Italy) and stored in a climatic chamber (KBF 240; Binder, Tuttlingen, Germany) to allow them to rise at 22°C and 80% relative humidity for 18 h to hydrolyze enzymatically aliquots of starches and proteins and obtain a more extensible and digestible structure (see Figure S2). The leavened loaves were sprinkled with a pinch of flour and then manually laminated under the pressure of both hands’ fingers from the center outward by turning the resulting disc several times. The final disc (i.e., the pizza base) had a diameter of approximately 28 ± 1 cm and an average mass of 250 ± 1 g. Such a base was baked as such (sample A) or garnished as shown in Table 1 (samples B–D).

**TABLE 1:** Samples of Neapolitan Pizza submitted to baking tests in the wood‐fired oven used in this study

Sample	Topping	Overall mass (g)
A	No garnishment	250 ± 1
B	Sunflower oil (30 g)	280 ± 2
C	Tomato puree (70 g)	320 ± 2
D	Tomato puree (70 g) and sunflower oil (30 g)	350 ± 3

### 2.3 Equipment

**FIGURE 1:**
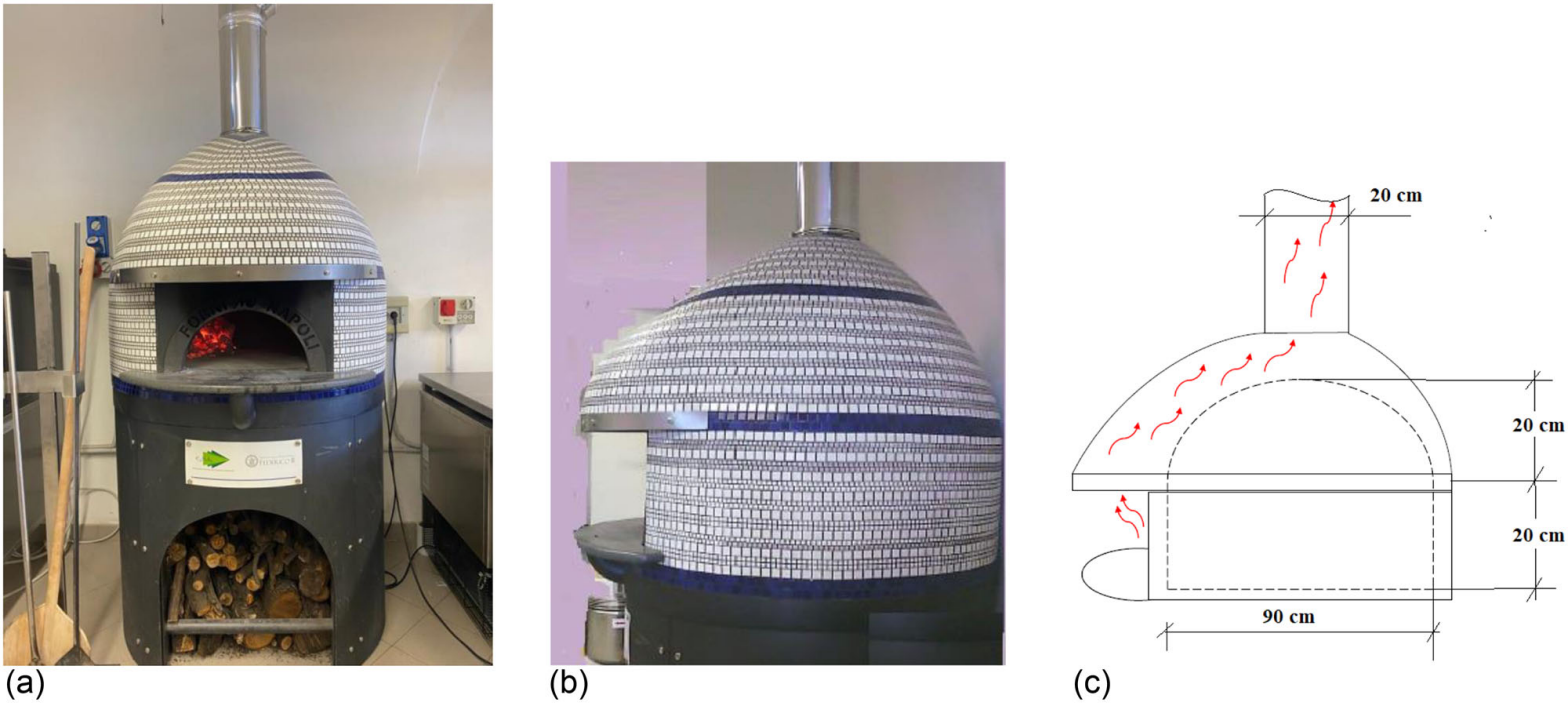
Front (a) and lateral (b) pictures of the wood‐fired pizza oven used in this study together with the geometry of its chamber (c)

Figure 1 shows the pilot‐scale wood‐fired pizza oven used in this study together with its chamber geometry. The oven chamber can be approximated to a cylinder with a diameter and height of 90 cm and 20 cm, respectively, surmounted by an oblate ellipsoidal vault of the same height. The pizza oven had a semicircular open mouth, and its diameter and height were equal to 44 and 22 cm, respectively. The oven walls and floor were approximately 10 cm thick. Oak logs were fed through the mouth of the pizza oven. As they were burning, the hot combustion flue gases were naturally drawn up and out of the chimney, while ambient air (at 36.4 ± 4.8°C and 20.4 ± 0.9% relative humidity) was sucked inside through the entry door. Approximately one‐fourth of the floor surface area was occupied by burning wood logs, while the remaining surface area was used for pizza baking.

### 2.4 Start‐up procedure

The start‐up procedure for this wood‐fired pizza oven was carried out as recommended by the manufacturer (MV Napoli Forni, Naples, Italy). The oven was fed with 1 kg of oak logs every 20 min (i.e., 3 kg/h) and fired for just 1 h on the first day (see Figure S3). Then, the same operation was repeated for 2 h on the second day, for 4 h on the third day, and finally for ∼8 h on the fourth day. During such lighting tests, the temperatures of the oven vault (*T*_V_) and floor (*T*_FL_) were monitored using a thermal imaging camera (FLIR E95 42°, FLIR System OU, Estonia) equipped with an uncooled microbolometer thermal sensor with dimensions of 7.888 × 5.916 mm and a resolution of 464 × 348 pixels. The pixel pitch of the sensor is 17 µm, the lens 10 mm and a field of view of 42° × 32°.

After this start‐up procedure, the wood‐fired pizza oven was retained as fully operative. In the circumstances, by feeding the oven with 3 kg of oak logs per hour (*Q*_fw_) for approximately 6 h, it was possible to stabilize the values of *T*_FL_ and *T*_V_, as reported below. Then, the firewood feed rate (*Q*_fw_) was varied from 3 to 9 kg/h to measure the responsiveness of the initial growth rate of *T*_FL_. Meanwhile, the mean superficial velocity (*v*_FG_) and temperature (*T*_FG_) of flue gases at the exit section of the oven chimney were simultaneously measured using a Hotwire Anemometer mod RS PRO RS‐8880 (RS Components, Corby, UK), while the flue gas temperature at the oven mouth was determined using a temperature logger 175 T3 (Testo SE & Co. KGaA, Titisee‐Neustadt, Germany). The fraction of wood logs that were effectively exploited to create heat during these trials was assessed by feeding the oven at each selected woodfire rate (*Q*_fw_) for approximately 6 h. One hour later, the residual unburned wood logs were separated from wood ashes and weighed to determine the mass of firewood effectvely burned. The combustion efficiency (η_comb_) was defined as the ratio between the mass of burned woodfire and the overall mass of oak logs supplied during each firing test. The combustion efficiency (*η*_comb_) was defined as the ratio between the mass of burned woodfire and the overall mass of oak logs supplied during each firing test.

### 2.5 Baking tests

Once the oven had been preheated at *Q*_fw_ = 3 kg/h for 6 h, the following tests were carried out in triplicate:

 (1) A circular aluminum tray (26 cm in diameter and 19.35 g in mass) was filled with 300 g of deionized water at an initial temperature of 25.8 ± 0.2°C, weighed, and then introduced into the oven, where it was kept for 10–80 s. As soon as the tray had been withdrawn from the oven, the temperature of the oven floor was suddenly measured in several areas different from that occupied by the tray using the above thermal imaging camera. Then, the mass of the water remaining in the tray and its temperature were measured using an analytical balance (Gibertini, Milano, Italy) and a temperature logger 175 T3 (Testo SE & Co. KGaA), respectively.
 (2) Each pizza sample of the four types shown in Table 1 was baked in the wood‐fired oven for 20, 40, 60, and 80 s. As soon as each sample was removed from the oven, the temperature of the oven floor area previously occupied by the sample itself, as well as that of the annular area around the sample itself, was measured as reported above. Then, as soon as the pizza sample had been extracted from the oven, the temperatures of the pizza disc in the rim and upper and lower central areas were measured using a thermal imaging camera. Finally, the sample mass was determined to assess its weight loss.

### 2.6 Energy performance assessment of the pizza oven

By neglecting the energy contribution of inlet air and firewood, the thermal performance of the pizza oven was assessed by writing the following heat balance:

(1) $$E_{\mathrm{fw}}=E_{S}+E_{W}+E_{\mathrm{FG}},$$

where *E*_fw_ is the energy supplied by firewood, *E*_S_ is the energy absorbed by the sample of choice, *E*_W_ is the energy lost by walls, and *E*_FG_ is the energy dissipated by flue gases.

The oak logs used here had moisture (*x_W_*) and ash (*x_A_*) contents of 5.67 ± 0.17 and 2.89 ± 0.66 g/100 g of wet matter, respectively. According to Vassilev et al. (2010), the dry matter of oak wood contains 50.6% carbon (*x*’*_C_*), 42.9% oxygen (*x*’*_O_*), 6.1% hydrogen (*x*’*_H_*), 0.3% nitrogen (*x*’*_N_*), and 0.1% sulfur (*x*’*_S_*). Thus, its higher (HHV) and lower (LHV) heating values were estimated as follows (Mukunda, 2009):

(2) $$\mathrm{HHV}=33.823\;x_{C}^{\prime }+144.249\left(x_{H}^{\prime }--x_{O}^{\prime }/8\right)+9.418\;x_{S}^{\prime },$$

(3) $$\mathrm{LHV}=\mathrm{HHV}--22.604\;x_{H}^{\prime }--2.581\;x_{M},$$

where *x*’*_C_*, *x*’*_H_*, *x*’*_O_*, and *x*’*_S_* are the weight fractions of carbon, hydrogen, oxygen, and sulfur on a dry basis of the biomass under study, and *x_M_* is the moisture content on wet matter. Thus, since HHV and LHV were approximately 18.19 and 16.66 MJ/kg, respectively, the energy supplied by oak logs was estimated as

(4) $$E_{\mathrm{fw}}=\eta_{\mathrm{comb}}Q_{\mathrm{fw}}\mathrm{LHV}\;t,$$

where *Q*_fw_ is the firewood feed rate (kg/h), *t* is the heating time (in hours), and *η*_comb_ is the combustion efficiency.

The energy stored by each sample, as such or including its vessel, upon heating from the initial temperature (*T*_S0_) to a generic temperature (*T*_S_) and the vaporization energy of the water lost were calculated as

(5) $$E_{S}=\left(m_{S}c_{\mathrm{ps}}+m_{V}c_{\mathrm{pV}}\right)\left(T_{S}-T_{S0}\right)+m_{\mathrm{ev}}\lambda_{\mathrm{ev}},$$

with

(6) $$m_{\mathrm{ev}}=m_{S0}-m_{S},$$

where *m*_S0_ and *m*_S_ are the initial and current masses of the sample, *m*_ev_ is the water evaporated, *m*_V_ is the mass of the vessel, *λ*_ev_ is the latent heat of water vaporization at *T*_S_ (in °C), and *c*_pS_ and *c*_pV_ are the specific heat values of the sample and vessel (in kJ kg^−1^ K^−1^).

The efficiency of the pizza oven (*η*_PO_) was estimated as the ratio between the energy absorbed by the load and that supplied by firewood (direct method):

(7) $$\eta_{\mathrm{PO}}=E_{S}/E_{\mathrm{fw}}.$$

Table 2 shows all the parameters used to calculate *η*_PO._

**TABLE 2:** Parameters used to estimate the thermal efficiency of the wood‐fired pizza oven during the water heating and baking tests performed in this study

Parameter	Value	Unit	References
Mass of water (*m*_S0_)	300.0 ± 0.1	g	
Mass of aluminum tray (*m*_V_)	19.35 ± 0.05	g	
Mass of pizza samples (*m*_S0_)	250–350	g	
Specific heat of water (*c*_PW_)	4.186	kJ kg^−1^ K^−1^	Singh et al. (2009)
Specific heat of aluminum tray (*c*_PV_)	0.890	kJ kg^−1^ K^−1^	Singh et al. (2009)
Specific heat of dough (*c*_PD_) or tomato puree (*c*_PT_) at *x*_W_	0.837 + 3.349 *x*_W_	kJ kg^−1^ K^−1^	Heldman and Lund (2007)
Specific heat of sunflower oil (*c*_PSO_)	(1.86 ± 0.03) + (2.25 ± 0.22) x10^−3^ *T*_S_	kJ kg^−1^ K^−1^	Santos et al. (2005)
Latent heat of water evaporation (*λ*_ev_)	$1.919\times 10^{3}{\left(\frac{T_{s}+273.15}{T_{s}+239.24}\right)}^{2}$	kJ kg^−1^	Henderson‐Sellers (1984)

### 2.7 Statistical analysis of data

Each baking test was carried out in triplicate. All parameters were shown as the average ± standard deviation (SD) and were analyzed by Tukey's test at a probability level (*p*) of 0.05. One‐way analysis of variance was carried out using SYSTAT version 8.0 (SPSS Inc., 1998).

## 3 RESULTS AND DISCUSSION

### 3.1 Start‐up procedure of the wood‐fired pizza oven

The start‐up procedure is aimed at controlling the intensity of the thermal reactions taking place during firing of the refractory bricks installed inside the wood‐fired pizza oven under study. In clay materials, such reactions may be either endothermic (due to dehydration process, change in crystal phase, or destruction of lattice structure) or exothermic (due to oxidation or new crystalline phase formation) (Grim & Johns, 1951). The loss of lattice water from the clay mineral components may be abrupt; thus, the heating rate is to be controlled to limit structural change and cause little or no disruption of the brick.

In this case, as suggested by the oven manufacturer, the oven was fired at a rate of 1 kg of firewood every 20 min for just 1 h on the first day, 2 h on the second day, 4 h on the third day and approximately 8 h on the fourth day.

**FIGURE 2:**
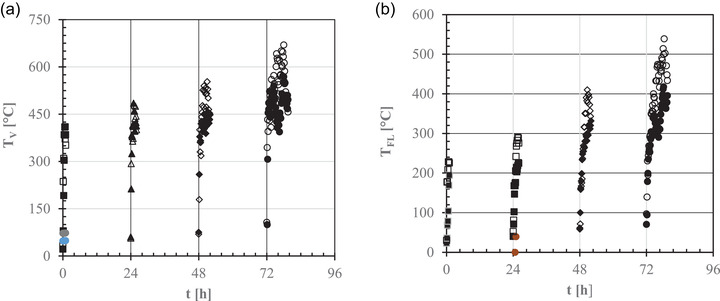
Time (*t*) course of the oven vault (*T*_V_: a) and floor (*T*_FL_: b) temperatures as measured using a thermal imaging camera during the first start‐up procedure (closed symbols) and the repeated one a week later (open symbols): ■, □, day 1; ▲, △, day 2; ◆, ◇, day 3; ●, ○, day 4

Figure 2 shows the time course of the temperatures of the oven vault (*T*_V_) and floor (*T*_FL_) during the start‐up procedure. A steep increase in both temperatures can be noted as a consequence of the heat released by burning logs. Moreover, as the heating time during each step was prolonged from 1 h to approximately 8 h, the initial values of *T*_V_ and *T*_FL_ tended to progressively increase due to the low thermal dispersivity of the insulated oven walls. As shown in Table S1, the initial mean values of the vault temperature gradient decreased from approximately 450°C/h to 340°C/h as the start‐up procedure progressed. In contrast, the initial derivate of the oven floor temperature with respect to time was approximately constant (148 ± 42°C/h).

**FIGURE 3:**
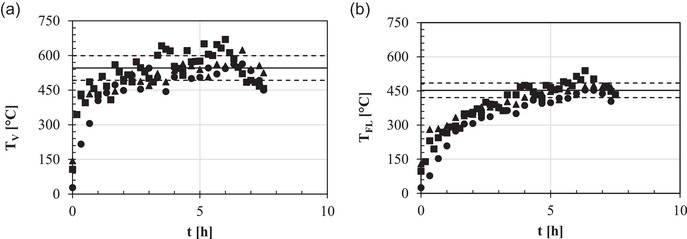
Time (*t*) course of the oven vault (*T*_V_: a) and floor (*T*_FL_: b) temperatures as measured using a thermal imaging camera during the lighting on the 11th (■), 22nd (●), and 23rd (▲) day: —, mean steady‐state temperature; —–, (mean ± SD) steady‐state temperature

Figure 3 shows the repeatability degree of the heating process of the pilot‐scale pizza oven when fed with 3 kg of oak logs per hour. Independent of the lighting frequency of the wood‐fired oven, after 4‐ to 6‐h firing, *T*_V_ or *T*_FL_ tended to a pseudosteady‐state value of 546 ± 53°C or 453 ± 32°C, respectively. Thus, all the following baking tests were performed on the condition that the pizza oven had been fired for not fewer than 6 h. Finally, how the initial growth rate of *T*_FL_ was affected by the firewood feed rate (*Q*_fw_) in the range of 3–9 kg/h was studied. Figure S4 shows the time course of *T*_FL_ at different *Q*_fw_ values. Regardless of *Q*_fw_, the oven floor temperature increased almost linearly with time, reached a maximum value, and then started to decline 30–40 min after firewood feeding had been stopped. For working times *t* ≤ 70 min, the increase in the oven floor temperature with respect to its initial value (*T*_FL_‐*T*_FLo_) was linearly related to the heating time (*t*), as indicated by the coefficients of determination (*r* ^2^) listed in Table 3.

**TABLE 3:** Mean and standard deviation (SD) values of the gradient of the oven floor temperature [(*dT*_FL_/*dt*)] and relative coefficient of determination (*r* ^2^) as a function of firewood feed rate (*Q*_fw_) used during a few lighting tests

*Q*_fw_	*dT*_FL_/*dt* (°C/h)	
(kg/h)	Mean ± SD	*r* ^2^
3.0	185 ± 3 ^a^	1.00
3.0	178 ± 29 ^a^	0.91
3.0	113 ± 4 ^b^	0.99
4.5	252 ± 20 ^c^	0.96
6.0	304 ± 25 ^d^	0.96
6.0	349 ± 13 ^d^	0.99
9.0	402 ± 35 ^e^	0.95
9.0	450 ± 50 ^e^	0.92
9.0	394 ± 41 ^e^	0.93
9.0	437 ± 42 ^e^	0.94

*Note*: Mean values of the oven floor temperature gradient followed by different superscript letters significantly differ by the Tukey test (*p* < 0.05).

**FIGURE 4:**
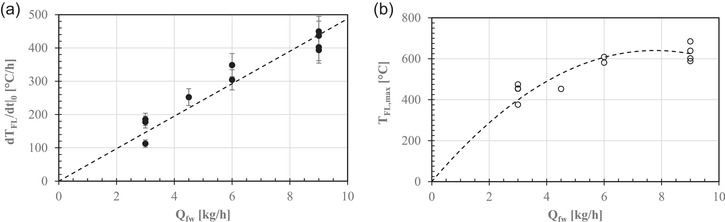
Effect of firewood feed rate (*Q*_fw_) on (a) the derivate of the oven floor temperature with respect to time (*dT*_FL_/*dt*|_0_) at *t* = 0 and (b) maximum oven floor temperature (*T*_FL, max_) in the wood‐fired pizza oven used here. Each broken line was plotted using Equation (8) or (9)

Figure 4a shows that the initial gradient of the oven floor temperature (*dT*_FL_/*dt*|_0_) was linearly related to *Q*_fw_ as

(8) $$\frac{dT_{\mathrm{FL}}}{dt}{|}_{0}=\left(49\pm 2\right)Q_{\mathrm{fw}}\;\left(r^{2}=0.99\right).$$

In contrast, the maximum value of the floor temperature (*T*_FL,max_) increased linearly for *Q*_fw_ <4 kg/h but tended to an asymptotic value of 629 ± 43°C for *Q*_fw_ = 9 kg/h (Figure 4b). Thus, a quadratic least squares regression was estimated to relate *T*_FL,max_ to *Q*_fw_:

(9) $$\begin{array}{ccc} T_{\mathrm{FL},\max} & = & \left(165\pm 11\right)Q_{\mathrm{fw}}-\left(10.6\pm 1.3\right){\left(Q_{\mathrm{fw}}\right)}^{2}\;\\ & & \left(r^{2}=0.99\right). \end{array}$$

Both Equations (8) and (9) might be used to control the thermal performance of the wood‐fired pizza oven.

**FIGURE 5:**
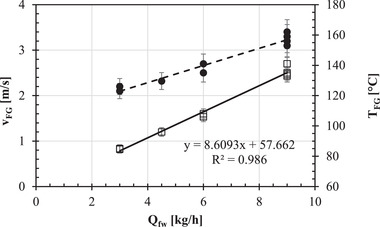
Effect of firewood feed rate (*Q*_fw_) on the mean superficial velocity (*v*_FG_: ●) and temperature (*T*_FG_: □) of flue gases at the exit section of the oven chimney. The broken or continuous line was plotted using Equation (10) or (11), respectively.

As the oak logs had been fed through the mouth of the pizza oven and had started to burn, the resulting hot combustion flue gases with a lower density than the outside air density were naturally forced to flow out of the oven chimney. Their effective volumetric flow rate is directly proportional to chimney height, temperature difference between the ascending flue gases and the outside air, and pressure drops along the chimney path (Rahman et al., 2021). Thus, as the woodfire feeding rate (*Q*_fw_) was increased from 3 to 9 kg/h, the increase in the temperature of flue gases lowered their density, which enhanced their volumetric flow rate. As shown in Figure 5, the mean superficial velocity (*v*_FG_) and temperature (*T*_FG_) of flue gases at the exit section of the oven chimney as measured using a Hotwire Anemometer were found to be almost linearly related for *Q*_fw_:

(10) $$v_{\mathrm{FG}}=\left(0.19\pm 0.02\right)\times Q_{\mathrm{fw}}+\left(1.5\pm 0.1\right)\;\left(r^{2}=0.954\right),$$

(11) $$T_{\mathrm{FG}}=\left(8.6\pm 0.4\right)\times Q_{\mathrm{fw}}+\left(57.7\pm 2.7\right)\;\left(r^{2}=0.986\right).$$

Finally, in a few burning tests carried out at *Q*_fw_ equal to 3 or 9 kg/h, the residual unburned wood logs amounted to approximately (13 ± 3) or (21 ± 4) percent of the overall mass of oak logs supplied, respectively. Thus, the combustion efficiency (*η*_comb_) tended to decrease from 87 ± 3% to 79 ± 4% as *Q*_fw_ was increased from 3 to 9 kg/h. Owing to the linear relationship between the other parameters characterizing the operation of the natural draft chimney of the wood‐fired pizza oven and firewood feed rate, *η*_comb_ is expected to decrease linearly from the above maximum and minimum values.

Such results might help unskilled operators to use the wood‐fired pizza oven in a quasi‐steady‐state regime when the woodfire feeding rate was varied from 3 to 9 kg/h.

### 3.2 Performance of the wood‐fired pizza oven

#### 3.2.1 Water heating test

Once the pilot‐scale wood‐fired pizza oven had been prelighted at *Q*_fw_ = 3 kg/h for 6 h, prefixed amounts of deionized water (300 g), as contained in aluminum circular trays having approximately the same diameter of a Neapolitan pizza, were heated for different times. Throughout such tests, the oven floor temperature was practically constant (448 ± 5°C). In contrast, the sample temperature (*T*_S_) increased from *T*_S0_ (25.8 ± 0.2°C) to 77.3 ± 1.2°C, while its mass (*m*_S_) decreased from 300 ± 0 g to 264 ± 4 g in just 80 s. Such data allowed the energy stored by the sample (*E*_S_) to be calculated using Equation (5) in conjunction with the thermal properties listed in Table 2. *E*_S_ was then referred to the energy generated by oak combustion, as calculated via Equation (4), to estimate the thermal efficiency of the pizza oven (*η*_PO_) using Equation (7).

Table 4 shows all the parameters either directly measured (*T*_FL_, *T*_S0_, *T*_S_, *m*_W_) or estimated (*E*_S_, *E*_fw_, *η*_PO_) as reported above.

**TABLE 4:** Main results (mean ± SD) of three repeated water heating tests performed in a wood‐fired pizza oven fed with 3 kg/h oak logs: Effect of time (*t*) on the oven floor temperature (*T*_FL_), initial (*T*_S0_) and current (*T*_S_) temperatures of water samples, instantaneous mass of water (*m*_W_), energy stored by the sample (*E*_S_), combustion heat (*E*_fw_), and oven efficiency (*η*_PO_)

*t*	*T*_FL_	*T*_S0_	*T*_S_	*m*_W_	*E*_S_	*E*_fw_	*η*_PO_
(s)	(°C)	(°C)	(°C)	(g)	(kJ)	(kJ)	(%)
0	‐	25.8 ± 0.2 ^a^	25.8 ± 0.2 ^a^	300.0 ± 0.1 ^a^	0.0	0	‐
10	447.0 ± 6.6 ^a^	25.8 ± 0.2 ^a^	44.3 ± 1.5 ^b^	298.0 ± 1.0 ^b^	28 ± 4	120.8	23.4 ± 3.5 ^a^
20	449.0 ± 1.7 ^a^	25.8 ± 0.3 ^a^	52.0 ± 1.0 ^c^	296.0 ± 1.7 ^c^	43 ± 5	241.6	17.6 ± 2.0 ^a,b^
30	449.3 ± 4.7 ^a^	25.8 ± 0.1 ^a^	58.7 ± 1.2 ^d^	293.0 ± 1.0 ^c^	58 ± 3	362.4	15.9 ± 1.0 ^b^
40	448.7 ± 6.0 ^a^	25.8 ± 0.1 ^a^	64.0 ± 1.0 ^e^	288.3 ± 2.3 ^c^	74 ± 6	483.2	15.4 ± 1.3 ^b^
50	446.0 ± 3.0 ^a^	25.8 ± 0.2 ^a^	70.7 ± 0.6 ^f^	285.0 ± 1.0 ^c^	90 ± 1	604.0	14.9 ± 0.2 ^b^
60	445.0 ± 3.0 ^a^	25.7 ± 0.2 ^a^	72.7 ± 0.6 ^g^	280.7 ± 1.5 ^d^	102 ± 4	724.8	14.0 ± 0.5 ^b,c^
70	449.7 ± 8.5 ^a^	25.7 ± 0.3 ^a^	75.7 ± 1.5 ^h^	269.3 ± 5.9 ^e^	129 ± 14	845.6	15.3 ± 1.6 ^b^
80	449.0 ± 7.0 ^a^	25.6 ± 0.4 ^a^	77.3 ± 1.2 ^h^	264.0 ± 3.6 ^e^	143 ± 8	966.4	14.8 ± 0.9 ^b^

*Note*: Mean values within the same parameter followed by different superscript letters significantly differ by the Tukey test (*p* < 0.05).

The average energy efficiency for the pizza oven examined here was equal to 14.7 ± 0.5%. This was in line with that of traditional domestic ovens but smaller than that estimated by Igo et al. (2020) for a metal fired‐wood oven. The thermal efficiency of well‐insulated conventional electric ovens usually ranges from 10% to 15%, while that of gaseous ovens varies from 6% to 7% because of the higher air flows and electric glow‐bar that run continuously to reignite the gas flame if it blows out (Barratt, 2021; Hager & Morawicki, 2013). Thus, the great majority of heat was lost by hot fumes or dispersed through the oven walls by convention or open oven mouth by radiation.

#### 3.2.2 Pizza baking tests

During such tests, white and tomato pizzas, as such or topped with sunflower oil, were baked for not more than 80 s in a preheated wood‐fired oven at *Q*_fw_ = 3 kg/h for 6 h.

Table 5 shows all the parameters directly measured, such as the temperature of the oven floor exposed to fire (T*_FL_*) or shielded by the pizza sample undergoing baking (*T*_FLbp_), temperatures of different pizza sectors, such as its rim (*T*_SR_) and upper (*T*_SU_) and lower (*T*_SL_) central areas, as well as the mass of sample (*m*_S_). Moreover, Table 5 lists the instantaneous values of other calculated parameters, such as the moisture mass fraction on an oil‐free basis (*x_W_*), energy stored by the sample (*E*_S_), combustion heat (*E*_fw_), and oven efficiency (*η*_PO_). Since the temperature of the pizza samples was generally not uniform throughout any test, its average temperature (*T*_S,ave_) was estimated by weighing the temperatures of the pizza sectors mentioned above on a mass basis by assuming that the rim, upper, and lower areas represented approximately 15%, 78%, and 7% of the overall sample mass, respectively. Moreover, the temperature of the areas topped with sunflower oil was used to calculate the sensible heat stored in the oil ingredient.

**TABLE 5:** Main results (mean ± SD) of three repeated baking tests performed in a wood‐fired pizza oven fed with 3 kg/h oak logs using four different pizza types: Effect of time (*t*) on the instantaneous temperature of the oven floor exposed to fire (*T*_FL_) or shielded by the pizza sample (*T*_FLbp_), temperatures of the pizza rim (*T*_SR_), upper (*T*_SU_) and lower (*T*_SL_) areas, mass of sample (*m*_S_), moisture fraction (*x*_W_), average sample temperature (*T*_S,ave_), energy stored by the sample (*E*_S_), combustion heat (*E*_fw_), and oven efficiency (*η*_PO_)

*t*	*T*_FL_	*T*_FLbp_	*T*_SR_	*T*_SU_	*T*_SL_	*m*_S_	*x*_W_	*T*_S,ave_	*E*_S_	*E*_fw_	*η*_PO_
(s)	(°C)	(°C)	(°C)	(°C)	(°C)	(g)	(g/g)	(°C)	(kJ)	(kJ)	(%)
White pizza											
0	442 ± 9 ^a^	442 ± 9 ^a^	21.0 ± 0.1 ^a^	21.0 ± 0.1 ^a^	21.0 ± 0.1 ^a^	250.0 ± 1.0 ^a^	0.450	21.0 ± 0.1 ^a^	0.0	0	–
20	441 ± 7 ^a^	363 ± 10 ^b^	80.0 ± 3.0 ^b^	103.0 ± 2.0 ^b^	84.0 ± 2.0 ^b^	248.2 ± 0.2 ^b^	0.446	98.5 ± 0.7 ^b^	48.9 ± 5.0 ^a^	241.6	20.2 ± 0.2 ^a^
40	436 ± 11 ^a^	348 ± 5 ^b^	116.0 ± 3.0 ^c^	138.0 ± 7.0 ^c^	97.0 ± 2.0 ^c^	245.9 ± 0.6 ^c^	0.440	131.8 ± 2.5 ^c^	72.4 ± 6.0 ^b^	483.2	15.0 ± 0.3 ^b^
60	435 ± 7 ^a^	332 ± 7 ^c^	130.0 ± 6.0 ^d^	157.0 ± 6.0 ^d^	102.0 ± 2.0 ^d^	243.0 ± 1.0 ^d^	0.434	149.2 ± 4.0 ^d^	87.1 ± 4.0 ^c^	724.8	12.0 ± 0.3 ^c^
80	432 ± 10 ^a^	325 ± 5 ^c^	148.0 ± 9.0 ^e^	182.0 ± 9.0 ^e^	106.0 ± 3.0 ^d^	240.6 ± 0.7 ^e^	0.428	171.5 ± 2.1 ^e^	103.5 ± 8.0 ^d^	966.4	10.7 ± 0.1 ^d^
White pizza garnished with sunflower oil											
0	446 ± 5 ^a^	448 ± 7 ^a^	21.0 ± 0.1 ^a^	21.0 ± 0.1 ^a^	21.0 ± 0.1 ^a^	280.0 ± 2.0 ^a^	0.450	21.0 ± 0.1 ^a^	0.0	241.6	–
20	443 ± 6 ^a^	351 ± 11 ^b^	86.0 ± 3.0 ^b^	100.0 ± 3.0 ^b^	81.0 ± 2.0 ^b^	278.4 ± 0.2 ^a^	0.446	97.0 ± 1.0 ^b^	52.3 ± 0.7 ^a^	483.2	21.6 ± 0.3 ^a^
40	441 ± 7 ^a^	342 ± 9 ^b^	116.0 ± 7.0 ^c^	128.0 ± 6.0 ^c^	93.0 ± 5.0 ^c^	276.7 ± 0.6 ^b^	0.442	124.0 ± 3.0 ^c^	72.8 ± 2.0 ^b^	724.8	15.1 ± 0.4 ^b^
60	439 ± 11 ^a^	327 ± 7 ^c^	149.0 ± 7.0 ^d^	148.0 ± 5.0 ^d^	101.0 ± 3.0 ^d^	272.4 ± 1.3 ^c^	0.432	145.0 ± 1.0 ^d^	93.8 ± 0.6 ^c^	966.4	12.9 ± 0.1 ^c^
80	434 ± 8 ^a^	314 ± 7 ^b,c^	169.0 ± 9.0 ^e^	156.0 ± 4.0 ^d^	105.0 ± 2.0 ^d^	267.7 ± 1.6 ^d^	0.421	155.0 ± 2.0 ^e^	108.1 ± 0.9 ^d^	241.6	11.2 ± 0.1 ^d^
Tomato pizza											
0	443 ± 8 ^a^	440 ± 7 ^a^	21.0 ± 0.1 ^a^	21.0 ± 0.1 ^a^	21.0 ± 0.1 ^a^	320.0 ± 2.0 ^a^	0.555	21.0 ± 0.1 ^a^	0.0	241.6	–
20	442 ± 7 ^a^	339 ± 10 ^b^	83.0 ± 2.0 ^b^	59.0 ± 2.0 ^b^	75.0 ± 2.0 ^b^	319.1 ± 0.3 ^a^	0.553	63.6 ± 1.4 ^b^	38.7 ± 1.2 ^a^	483.2	16.0 ± 0.5 ^a^
40	439 ± 7 ^a^	328 ± 6 ^b^	113.0 ± 4.0 ^c^	71.0 ± 2.0 ^c^	92.0 ± 3.0 ^c^	317.1 ± 0.5 ^b^	0.551	79.0 ± 0.8 ^c^	56.1 ± 0.6 ^b^	724.8	11.6 ± 0.1 ^b^
60	438 ± 8 ^a^	320 ± 10 ^b,c^	124.0 ± 3.0 ^d^	76.0 ± 2.0 ^d^	96.0 ± 2.0 ^c^	314.1 ± 0.3 ^c^	0.546	84.8 ± 1.1 ^d^	67.2 ± 0.9 ^c^	966.4	9.3 ± 0.1 ^c^
80	436 ± 6 ^a^	304 ± 5 ^c^	136.0 ± 3.0 ^e^	81.0 ± 2.0 ^e^	101.0 ± 2.0 ^d^	311.2 ± 0.8 ^d^	0.542	90.6 ± 0.4 ^e^	77.9 ± 0.3 ^d^	241.6	8.1 ± 0.1 ^d^
Tomato pizza garnished with sunflower oil											
				Tomato area Oil area							
0	440 ± 7 ^a^	438 ± 10 ^a^	21.0 ± 0.1 ^a^	21.0 ± 0.1 ^a^ 21.0 ± 0.1^a^	21.0 ± 0.1 ^a^	350.0 ± 3.0 ^a^	0.555	21.0 ± 0.1 ^a^	0.0	241.6	–
20	438 ± 5 ^a^	332 ± 12 ^b^	88.0 ± 3.0 ^b^	61.0 ± 3.0 ^b^ 89.0 ± 5.0^b^	74.0 ± 3.0 ^b^	349.4 ± 0.1 ^a^	0.554	66.3 ± 2.6 ^b^	44.5 ± 2.5 ^a^	483.2	18.4 ± 1.0 ^a^
40	437 ± 7 ^a^	318 ± 5 ^b,c^	115.0 ± 5.0 ^c^	73.0 ± 2.0 ^c^ 100.0 ± 4.0^c^	87.0 ± 2.0 ^c^	347.2 ± 0.5 ^b^	0.551	80.3 ± 0.1 ^c^	62.0 ± 0.1 b	724.8	12.8 ± 0.1 ^b^
60	437 ± 6 ^a^	313 ± 7 ^b,c^	128.0 ± 5.0 ^d^	79.0 ± 2.0 ^d^ 103.0 ± 2.0^c^	93.0 ± 2.0 ^d^	344.7 ± 0.3 ^c^	0.547	87.3 ± 0.6 ^d^	73.2 ± 0.5 ^c^	966.4	10.1 ± 0.1 ^c^
80	436 ± 6 ^a^	309 ± 7 ^c^	141.0 ± 2.0 ^e^	84.0 ± 2.0 ^e^ 106.0 ± 2.0^c^	102.0 ± 2.0 ^e^	341.0 ± 1.9 ^d^	0.542	94.0 ± 0.5 ^e^	86.5 ± 0.5 ^d^	241.6	9.0 ± 0.1 ^d^

*Note*: Mean values within the same parameter at different baking times followed by different superscript letters significantly differ by the Tukey test (*p* < 0.05).

First, during all such tests, the wood‐fired oven behaved in almost quasi‐steady‐state conditions, and its floor temperature showed no statistically significant variation at approximately 439 ± 8°C at the probability level of 0.05. Second, the moisture content on an oil‐free basis (*x_W_*) of white pizza samples decreased from 0.45 to 0.42 g/g, while that of tomato pizza samples decreased from 0.56 to 0.54 g/g. The temperature of the upper central areas of white pizza samples tended to the smoke point (∼211°C) of sunflower oil at ambient pressure (http://www.centrafoods.com/blog/edible‐oil‐smoke‐flash‐points‐temperature‐chart), whereas that of the tomato pizza counterparts increased to a value well below the boiling of water, that is 82–84°C (Table 5). In contrast, owing to its direct contact with the oven floor, the lower side of each sample rapidly reached a temperature more (105–106°C) or less (101–102°C) greater than the water boiling point depending on its smaller or greater moisture content, respectively. When topped with oil, each pizza sample stored a greater amount of energy, that is, 108 instead of 104 kJ in the case of white pizza or 87 vs. 78 kJ in the case of tomato pizza (Table 5). It can be noted that the specific energy stored by pizza samples decreased almost linearly (*r* ^2^ = 0.88) from 430 ± 5 to 254 ± 1 kJ/kg as the mass of the garnished pizza sample increased from 0.25 to 0.35 kg. Since the pizza oven was operating in pseudosteady‐state conditions, the net heat flux transferred to each pizza sample by radiation and convention was, in all probability, approximately constant and almost insensitive to the emissivity of the different pizza topping ingredients used (Ciarmiello & Morrone, 2016b). Thus, despite the difference in the thermal properties (including emissivity) of the pizza topping ingredients, the increase in the temperature of each pizza sample was inversely proportional to its overall mass. Finally, the oven efficiency was not significantly different at the 95% confidence level when baking white pizza as such (14.5 ± 3.8%), and white (15.2 ± 4.1%) and tomato pizzas (12.6 ± 3.8%) were both topped with sunflower oil. The thermal efficiency was reduced to 11.2 ± 3.2% in the case of tomato pizza, which was significantly different from the above values at the probability level of 0.05. Altogether, the average thermal efficiency of the wood‐fired oven examined in this study was approximately 13 ± 4% when referring to both the water heating and baking tests mentioned above. Obviously, such an efficiency is to be regarded as overestimated, since it accounts for the only combustion energy freed during the baking tests and neglects the energy supplied by firewood during the preliminary 6‐h prelighting step needed to put the oven in quasi pseudo‐steady state conditions.

In the circumstances, despite the high quality of baking provided by such equipment, its use results not only in excessive consumption of biomass fuels, leading to natural forest degradation and deforestation, especially in a few areas of Africa (Okino et al., 2021) but also in high indoor levels of air pollutants (i.e., carbon monoxide, polycyclic aromatic hydrocarbons, sulfur dioxide, nitrogen oxide, black carbon, and particulate matter), as observed in several metropolitan areas (Apurva, 2016; Kumar et al., 2016) and in a study dealing with the environmental profile of a few household cooking systems, including firewood ones (Cimini & Moresi, 2022).

To surmount such problematic issues, the Associazione Verace Pizza Napoletana (AVPN, 2004) would allow the use of an alternative electric oven (i.e., the *Scugnizzo Napoletano* oven developed by Izzo Forni, Naples, Italy: https://www.izzoforni.it/izzonapoletano/), since such an oven succeeded in a series of physical and sensory tests, as well as numerical tests using a three‐dimensional computational fluid dynamics numerical model under unsteady and steady conditions (Ciarmiello & Morrone, 2016b).

## 4 CONCLUSIONS

In this study, the performance of a pilot‐scale wood‐fired pizza oven similar to those commonly used in Neapolitan pizzerias in Italy was assessed. First, its start‐up procedure was performed. Second, it was studied how, independent of the operator's ability, the oven can be put in quasi‐steady‐state conditions with its dome and floor temperatures exhibiting no appreciable fluctuations by varying the firewood feed rate from 3 to 9 kg/h. Third, two different baking tests were carried out using either just water or four pizza types as such or topped with tomato puree and/or sunflower oil. In both tests, the thermal efficiency was approximately 13% of the energy supplied by oak log burning. In these circumstances, the use of such equipment leads to an inefficient use of wood as well as poor indoor and outdoor air quality. Further work should be aimed at modeling the time course of the heat transferred via radiation, convention, and conduction to each pizza under baking.

## AUTHOR CONTRIBUTIONS

Conceptualization, investigation, validation, and writing—review and editing: Aniello Falciano. Conceptualization, funding acquisition, investigation, project administration, validation, and writing—review and editing: Paolo Masi. Conceptualization, formal analysis, investigation, methodology, software, writing – original draft, writing—review and editing: Mauro Moresi. All authors have read and agreed to the published version of the manuscript.

## CONFLICT OF INTEREST

The authors declare no competing interest.

## Supporting information

Figure S1. Picture showing dough preparation in a spiral mixer.
Figure S2. Picture showing a few dough balls placed over a plastic tray at the (a) beginning and (b) end of the bulk fermentation.
Figure S3. Pictures of the wood‐fired oven with wood logs burning as viewed altogether (a) or from its mouth (b).
Figure S4. Time (t) course of the oven floor (T_FL_) temperature, as measured using a thermal imaging camera, at different firewood feed rates (Q_fw_): ⬤, Q_fw_ = 3 kg/h; ▴, Q_fw_ = 4.5 kg/h; ⬥, ◇, Q_fw_ = 6 kg/h; ⬜ , ⬛, ⬜ , ⬜ , Q_fw_ = 9 kg/h.
Table S1. Mean and standard deviation (SD) values of the initial temperature gradients of the oven vault (dT_V_/dt) and floor (dT_FL_/dt) and relative coefficient of determination (r^2^) during the 4‐day start‐up procedure and that repeated a week later.

‐kg/h.

## Data Availability

The datasets generated during and/or analyzed during the current study are available from the corresponding author on reasonable request.
