# Supplementary material for: Performance characterization of a traditional wood‐fired pizza oven
Source: J Food Sci. 2022 Aug 7;87(9):4107–18. doi: 10.1111/1750-3841.16268 (PMC9804529; doi:10.1111/1750-3841.16268)
Supplement: Supplementary file 1 — Figure S1. Picture showing dough preparation in a spiral mixer. Figure S2. Picture showing a few dough balls placed over a plastic tray at the (a) beginning and (b) end of the bulk fermentation. Figure S3. Pictures of the wood‐fired oven with wood logs burning as viewed altogether (a) or from its mouth (b). Figure S4. Time (t) course of the oven floor (TFL) temperature, as measured using a thermal imaging camera, at different firewood feed rates (Qfw): ⬤, Qfw = 3 kg/h; ▴, Qfw = 4.5 kg/h; ⬥, ◇, Qfw = 6 kg/h; ⬜ , ⬛, ⬜ , ⬜ , Qfw = 9 kg/h. Table S1. Mean and standard deviation (SD) values of the initial temperature gradients of the oven vault (dTV/dt) and floor (dTFL/dt) and relative coefficient of determination (r2) during the 4‐day start‐up procedure and that repeated a week later. [file JFDS-87-4107-s001.docx]

**ELECTRONIC SUPPLEMENT**

**Figure S1** Picture showing dough preparation in a spiral mixer.

**Figure S2** Picture showing a few dough balls placed over a plastic tray at the (**a**)

beginning and (**b**) end of the bulk fermentation.

1. **b)**

**Figure S3** Pictures of the wood-fired oven with wood logs burning as viewed altogether (**a**) or from its mouth (**b**).

**a) b)**

**Figure S4** Time (t) course of the oven floor (T_FL_) temperature, as measured using a thermal imaging camera, at different firewood feed rates (Q_fw_): ●, Q_fw_=3 kg/h; ▲, Q_fw_=4.5 kg/h; ⯁, ◇, Q_fw_=6 kg/h; □, ■, □, □, Q_fw_=9 kg/h.

**Table S1** Mean and standard deviation (sd) values of the initial temperature gradients of the oven vault (dT_V_/dt ) and floor (dT_FL_/dt) and relative coefficient of determination (r^2^) during the 4-day start-up procedure and that repeated a week later.

| **Start-up step** | **dT_V_/dt [°C/h]** | **r^2^** | **dT_FL_/dt [°C/h]** | **r^2^** |
| --- | --- | --- | --- | --- |
|  | mean ± sd |  | mean± sd |  |
| Day 1 | 456 ± 36 ^A^ | 0.96 | 155 ± 11 ^a^ | 0.97 |
| Day 1 bis | 450± 68 ^A^ | 0.88 | 260± 38 ^b^ | 0.89 |
| Day 2 | 334± 42 ^B^ | 0.88 | 141± 9 ^c^ | 0.96 |
| Day 2 bis | 346± 43 ^B^ | 0.88 | 148 ± 9 ^a,c^ | 0.96 |
| Day 3 | 309± 40 ^C^ | 0.87 | 140 ±10 ^c^ | 0.94 |
| Day 3 bis | 342 ± 31 ^B^ | 0.93 | 135 ±5 ^c^ | 0.98 |
| Day 4 | 361± 50 ^B^ | 0.85 | 145 ± 10 ^c^ | 0.94 |
| Day 4 bis | 323 ± 49 ^B,C^ | 0.83 | 114± 6 ^d^ | 0.95 |

Different uppercase and lowercase Latin letters indicate statistically significant difference among the temperature gradient means during each step of the start-up procedure used at the probability level of 0.05.
