# Supplementary material for: Performance characterization of a traditional wood‐fired pizza oven
Source: J Food Sci. 2022 Aug 7;87(9):4107–18. doi: 10.1111/1750-3841.16268 (PMC9804529; doi:10.1111/1750-3841.16268)
Supplement: Supplementary file 2 — ‐kg/h. [file JFDS-87-4107-s002.pdf]

ELECTRONIC SUPPLEMENT

Figure S1 Picture showing dough preparation in a spiral mixer.

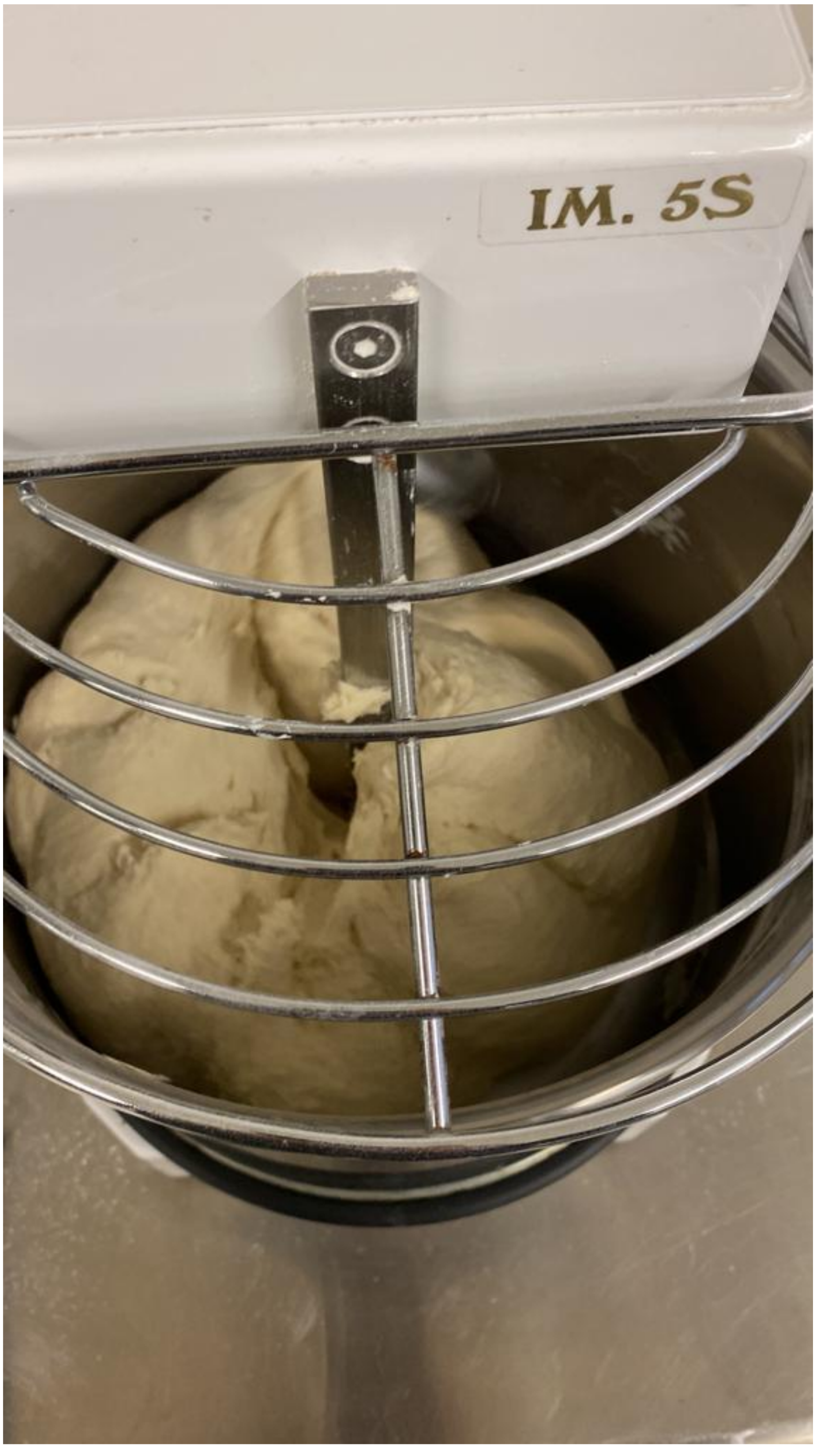

Figure S2 Picture showing a few dough balls placed over a plastic tray at the (**a**) beginning and (**b**) end of the bulk fermentation.

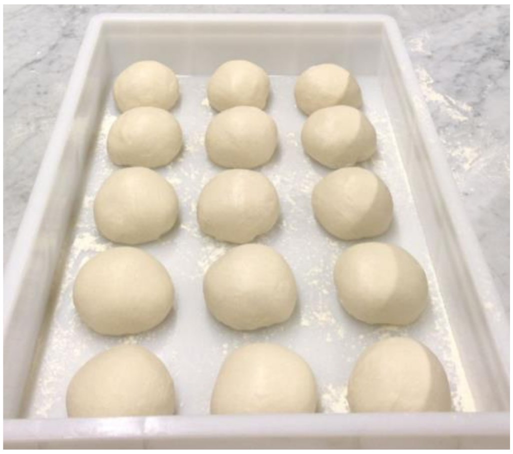

a)

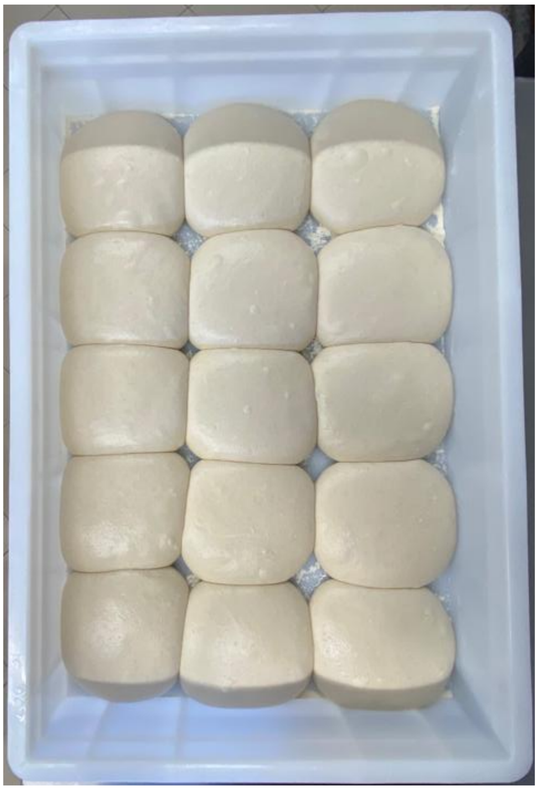

b)

Figure S3 Pictures of the wood-fired oven with wood logs burning as viewed altogether (**a**) or from its mouth (**b**).

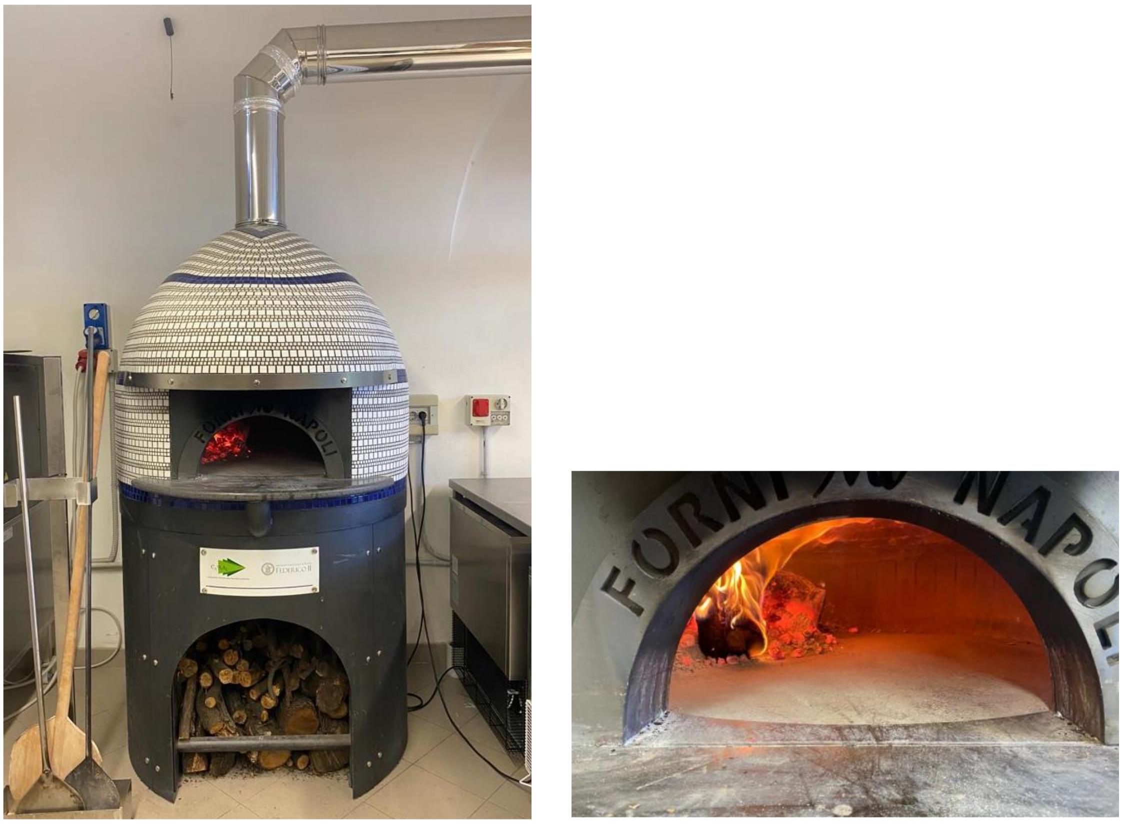

a) **b)**

Figure S4 Time (t) course of the oven floor (T_{FL}) temperature, as measured using a thermal imaging camera, at different firewood feed rates (Q_{fw}): ●, Q_{fw}=3 kg/h; ▲, Q_{fw}=4.5 kg/h; ◆, ◇, Q_{fw}=6 kg/h; □, ■, □, □, Q_{fw}=9 kg/h.

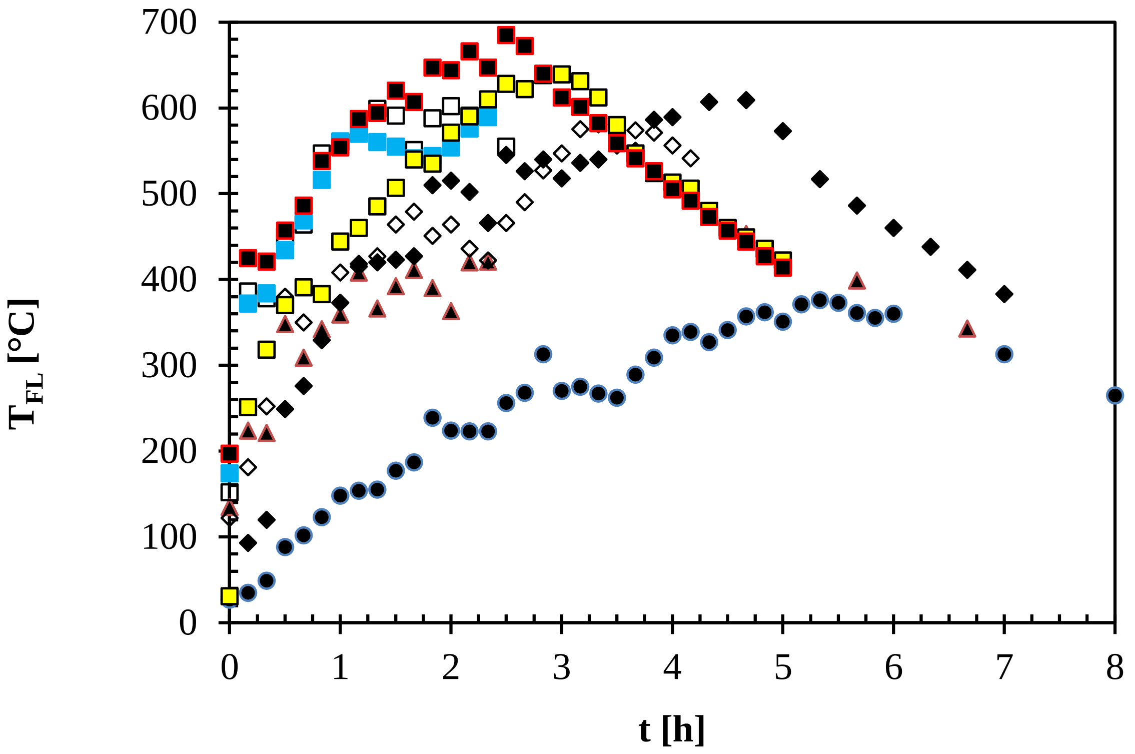

Table S1 Mean and standard deviation (sd) values of the initial temperature gradients of the oven vault (dT_V/dt) and floor (dT_{FL}/dt) and relative coefficient of determination (r^2) during the 4-day start-up procedure and that repeated a week later.

**Start-up step**	**dT_V/dt [°C/h]**	**r^2**	**dT_{FL}/dt [°C/h]**	**r^2**
	mean ± sd		mean± sd	
Day 1	456 ± 36 ^{A}	0.96	155 ± 11 ^{a}	0.97
Day 1 bis	450± 68 ^{A}	0.88	260± 38 ^{b}	0.89
Day 2	334± 42 ^{B}	0.88	141± 9 ^{c}	0.96
Day 2 bis	346± 43 ^{B}	0.88	148 ± 9 ^{a,c}	0.96
Day 3	309± 40 ^{C}	0.87	140 ±10 ^{c}	0.94
Day 3 bis	342 ± 31 ^{B}	0.93	135 ±5 ^{c}	0.98
Day 4	361± 50 ^{B}	0.85	145 ± 10 ^{c}	0.94
Day 4 bis	323 ± 49 ^{B,C}	0.83	114± 6 ^{d}	0.95

Different uppercase and lowercase Latin letters indicate statistically significant difference among the temperature gradient means during each step of the start-up procedure used at the probability level of 0.05.
